# Acupuncture Improves Sleep Disorders and Depression among Patients with Parkinson’s Disease: A Meta-Analysis

**DOI:** 10.3390/healthcare11142042

**Published:** 2023-07-17

**Authors:** Wei-Ti Hsu, Chieh-Min Hsu, Shao-Chi Hung, Shih-Ya Hung

**Affiliations:** 1 Graduate Institute of Biomedical Sciences, China Medical University, Taichung 40402, Taiwan; u108305203@cmu.edu.tw; 2 Department of Anesthesiology, China Medical University Hospital, Taichung 40447, Taiwan; d17060@mail.cmuh.org.tw (C.-M.H.); d31232@mail.cmuh.org.tw (S.-C.H.); 3 Graduate Institute of Acupuncture Science, China Medical University, Taichung 40402, Taiwan; 4 Division of Surgery, Department of Medical Research, China Medical University Hospital, Taichung 40447, Taiwan

**Keywords:** acupuncture, depression, non-motor symptoms, Parkinson’s disease, sleep disorders

## Abstract

Parkinson’s disease (PD) is associated with a range of non-motor symptoms that lack effective treatments. Acupuncture is a popular alternative therapy for PD patients that has been shown to improve motor symptoms. However, the efficacy of acupuncture in treating non-motor symptoms has remained controversial. The goal of our study was to systematically assess the existing evidence for acupuncture’s efficacy in treating PD non-motor symptoms of sleep disorders, depression, anxiety, and fatigue. We conducted a meta-analysis of clinical trials by searching Pubmed, Embase, CINAHL, and Web of Science as electronic databases to evaluate acupuncture treatment for PD non-motor symptoms. Thirteen clinical trials met our inclusion criteria, and their methodological quality was assessed using the modified Jadad scale, indicating a moderate overall quality. Our results showed that acupuncture improved PD-related sleep disorders and depression but had no effect on anxiety and fatigue. Our meta-analysis suggests that acupuncture can be used as a complementary treatment for sleep disturbances and depression in PD patients and may exhibit a dual therapeutic effect on motor and non-motor symptoms. However, further well-designed clinical trials with larger sample sizes are needed to confirm these findings. Overall, our study highlights the potential of acupuncture as a viable complementary therapy for the treatment of PD non-motor symptoms of sleep disorders and depression, which can improve the quality of life of PD patients.

## 1. Introduction

Parkinson’s disease (PD) is a common chronic and progressive neurodegenerative disorder that affects motor function but also leads to a range of non-motor symptoms [1]. Pathological hallmarks of PD are associated with early prominent death of dopaminergic neurons in the substantia nigra pars compacta (SNpc) for an unknown reason resulting in dopamine deficiency within the striatum and leading to classical parkinsonian motor symptoms [2,3]. The motor symptoms of PD are characterized by bradykinesia, rigidity, tremor, and postural instability; non-motor symptoms are characterized by depression, anxiety, cognitive impairment, sleep disturbances, and others [2]. Non-motor symptoms are common in patients with PD and can appear before motor features and progress in severity and diversity as the disease evolves [4]. While current treatments such as levodopa (as dopamine replacement therapy), dopamine agonists, and deep brain stimulation exert good control for PD motor symptoms, they do not effectively address non-motor symptoms. Therefore, developing new treatments for non-motor symptoms remains a priority in PD research.

Acupuncture is a traditional Chinese medicine technique that has been used for the treatment of neurodegenerative diseases such as Alzheimer’s disease and PD [5]. Electroacupuncture, a form of acupuncture that has been increasingly popular since the 1970s, is preferred among researchers due to its ease of standardization [6]. In PD patients, acupuncture is among the top three most popular alternative therapies [7]. Acupuncture has been reported to have therapeutic effects on both motor and non-motor symptoms. For motor symptoms, a clinical trial has shown that bee venom acupuncture or acupuncture treatments can significantly improve motor symptoms in idiopathic PD patients [8]. Electroacupuncture treatment has also been found to improve rigidity, balance, and gait in PD patients [9,10]. Additionally, a recent multicenter randomized controlled trial demonstrated that electroacupuncture combined with conventional pharmacological treatment significantly enhances motor function in PD patients [11]. The neural mechanism underlying the effect of acupuncture on the motor function of PD patients has been investigated using functional magnetic resonance imaging (fMRI), which shows that acupuncture stimulation at GB34 acupoint can activate the putamen and primary motor cortex, improving motor function [12]. Our animal studies also support the therapeutic potential of electroacupuncture in PD, demonstrating that electroacupuncture at GB34 and LR3 acupoints promotes motor function recovery and reduces dopaminergic neuron degeneration via Akt-BDNF pathway and autophagy activation in the substantia nigra and striatum, respectively [6,13]. These findings suggest that acupuncture may have a promising role in the treatment of PD motor symptoms through modulation of neuronal activity in specific brain regions.

PD patients frequently experience non-motor symptoms. A study of 402 PD patients published in 2021 found non-motor symptoms present in 99.7% of them, with higher PD severity associated with a greater prevalence of non-motor symptoms [14]. Among non-motor symptoms, sleep disturbances are the most common, affecting 60% to 90% of patients with various sleep disorders, such as insomnia, sleep-disordered breathing disorders, and excessive daytime sleepiness [15]. Rates of depression, anxiety, and fatigue in PD patients are highly variable across studies, with some finding prevalence rates as low as 2.7% and others finding rates exceeding 90% [16,17,18]. While acupuncture is a popular alternative therapy for PD patients that has been shown to improve motor symptoms, its efficacy in treating non-motor symptoms, such as sleep disorders, depression, anxiety, and fatigue, remains controversial. Some studies suggest that acupuncture can improve sleep efficiency, depression, and anxiety in PD patients. For example, Li et al. (2022) showed that acupuncture led to increased sleep efficiency in a dose-dependent manner compared to sham acupuncture in PD patients [19]. However, other studies have produced inconclusive results. For instance, a clinical trial found that acupuncture for six weeks was not effective in improving sleep quality and excessive daytime somnolence compared with sham acupuncture in PD patients [20]. A meta-analysis of 27 randomized controlled trials suggests that acupuncture treatment could ameliorate the symptoms of depression, quality of life, cognition, total mentation, behavior and mood, and activities of daily living in PD patients [21]. Moreover, a randomized, double-blinded clinical trial found that real acupuncture for eight weeks improves anxiety in PD patients at the end of an 8-week follow-up period [22]. Additionally, a randomized clinical trial found that real and sham acupuncture have equal efficacy in improving PD-related fatigue [23]. The report suggests that the treatment’s benefit of acupuncture may be due to placebo or other non-specific effects.

Acupuncture is a popular complementary therapy for PD patients. While it has been shown to improve motor symptoms, its effect on non-motor symptoms remains limited and inconsistent. Given the lack of effective treatments for PD non-motor symptoms and the limited understanding of their underlying neuroanatomical and neuropharmacological bases, it is essential to evaluate the current evidence for using acupuncture to manage these symptoms. To address this gap, we conducted a meta-analysis of published clinical trials to evaluate the efficacy of acupuncture in improving non-motor symptoms in PD patients. The primary endpoint of our study is the effect of acupuncture on sleep quality, while the secondary endpoints are its impact on depression, anxiety, and fatigue. By providing a comprehensive summary of the existing evidence, our study aims to inform clinical practice and improve the quality of life for PD patients with non-motor symptoms. Additionally, our study may provide insights into whether acupuncture has dual treatment benefits for both motor and non-motor symptoms in PD patients.

## 2. Materials and Methods

This study is a systematic review and meta-analysis based on a protocol (PROSPERO ID: CRD42022354083) and followed the Cochrane Handbook for Systematic Reviews of Interventions [24]. Institutional Review Board (IRB) approval was not required since all the data analyzed were extracted from public literature.

### 2.1. Search Strategy

We searched the electronic databases of PubMed, Embase, CINAHL, and Web of Science for clinical trials on acupuncture for non-motor symptoms in patients with PD from the inception date to 21 June 2023, in accordance with the Preferred Reporting Items for Systematic Reviews and Meta-Analyses (PRISMA) statement [25]. Two authors (Hsu CH and Hung SC) independently searched for potential literature using a combination of keywords such as “Parkinson’s disease”, “Parkinsonism”, “acupuncture”, and “electroacupuncture”. Literature search strings for our meta-analysis in various databases are shown in the Supplementary Materials (Table S1). Additionally, we searched for relevant review articles to identify further studies.

### 2.2. Inclusion and Exclusion Criteria

We screened the titles and abstracts of all the identified literature and included studies that met the following criteria: (1) human-controlled trials; (2) participants diagnosed with Parkinson’s disease or Parkinsonism; (3) intervention group receiving acupuncture or electroacupuncture; (4) outcome assessment included non-motor symptoms of Parkinson’s disease; and (5) control group with sham acupuncture or no acupuncture. We excluded studies that did not meet the inclusion criteria, such as review articles, protocols, conference papers, case reports, letters, and editorials, and those that combined acupuncture with other complementary and alternative treatments, such as exercise and aromatherapy. Two authors (Hsu WT and Hsu CH) assessed the full texts of selected literature for meta-analysis, and the advising professor, Hung SY, resolved any disagreements.

### 2.3. Data Extraction Process

To extract relevant clinical information and research-related data, two authors (Hsu WT and Hsu CH) independently collected the following details from the selected studies: (1) first author, publication year, country, and study design; (2) intervention type and participant age; (3) the number of participants randomized to each trial arm; (4) participant population and average disease duration; (5) diagnostic criteria for non-motor symptoms; (6) outcome of non-motor symptoms, including treatment duration and rating scales for sleep, depression, anxiety, and fatigue, along with statistical data such as sample size, mean, and standard deviation. In case of any conflicts during the data extraction process, the advising professor Hung SY was consulted to resolve them.

### 2.4. Quality Assessment of the Included Studies

The methodological quality of all included studies was independently assessed by two authors (Hsu WT and Hsu CH) using the modified Jadad scale [26,27]. This scale includes eight items to evaluate randomization, blinding, withdrawals, dropouts, inclusion and exclusion criteria, adverse effects, and statistical analysis. The total score for each study ranges from 0 to 8, with a score of less than 3 indicating low quality, 4–6 indicating moderate quality, and a score of 7 or 8 indicating high quality. In case of any disagreements during the quality assessment, the advising professor Hung SY was consulted to resolve them.

### 2.5. Statistical Analysis Methods

Comprehensive Meta-Analysis (CMA version 3) was used to process all meta-analyses. For continuous outcomes such as rating scales for sleep, depression, anxiety, and fatigue, we used the standardized mean difference (SMD) or mean difference (MD) with 95% confidence intervals (CI) to analyze effect sizes (ESs) due to different units and rating scales used in various studies. An ES analysis with a *p*-value of less than 0.05 was considered statistically significant. We used the random-effect model to estimate the pooled effect size, and the *I*-square (*I*^2^) statistics assessed heterogeneity. Subgroup meta-analysis was used to investigate the potential sources of substantial heterogeneity between studies (*I*^2^ > 50%) or different rating scales [24]. We conducted a subgroup meta-analysis to investigate the effect of different interventions such as acupuncture, bee venom, or electroacupuncture. We also performed a subgroup meta-analysis to investigate whether different control groups, such as sham acupuncture or no acupuncture, affected the outcomes’ effect.

## 3. Results

### 3.1. Identification and Selection of Studies

**Figure 1:**
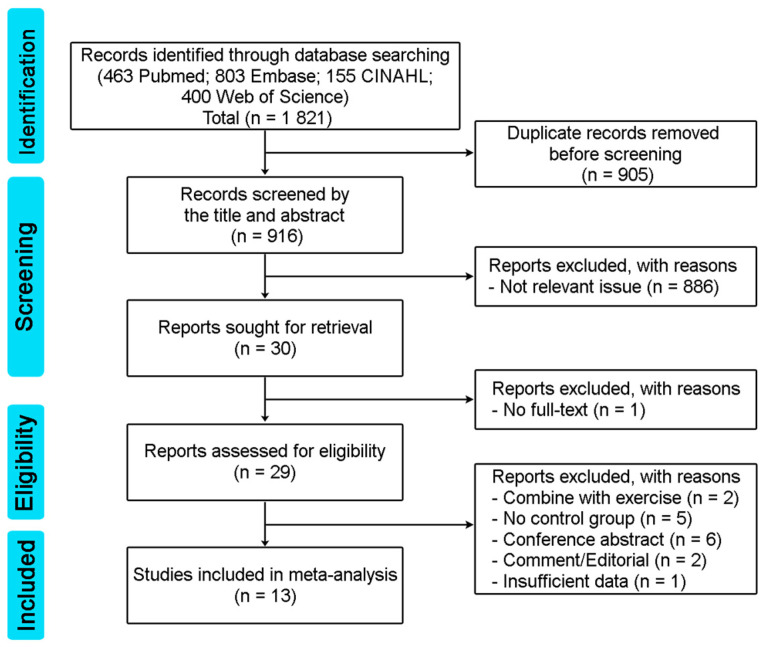
Search flow diagram according to PRISMA guideline.

Initially, a total of 1821 articles were identified through database searches, including 463 from Pubmed, 803 from Embases, 155 from CINAHL, and 400 from Web of Science. After removing 905 duplicate articles, 916 articles underwent title and abstract screening. Out of these, 886 articles were excluded due to their lack of relevance, resulting in 30 articles that met the inclusion criteria. One of the 30 articles was excluded due to a lack of full text, leaving 29 eligible articles. Sixteen articles were then excluded for the following reasons: combined with exercise (2 articles), no control group (5 articles), conference abstract (6 articles), comment/editorial (2 articles), and insufficient data (1 article). Finally, 13 articles were included in the meta-analysis [8,19,20,22,23,28,29,30,31,32,33,34,35]. Figure 1 depicts the screening process in a flow diagram.

### 3.2. Study Characteristics and Patient Populations

The 13 studies included in this meta-analysis had a total of 630 participants, and their characteristics are presented in Table 1. All studies were published between 2012 and 2022, and the geographical distribution of studies was as follows: two studies from Korea [8,32], six from China [19,22,28,30,34,35], two from Taiwan [29,33], one from the USA [20], one from Brazil [31], and one from Singapore [23]. Of the 13 studies, 9 studies used acupuncture or bee venom as the main intervention, while the remaining 4 studies used electroacupuncture [28,30,34,35]. Four studies used sham acupuncture as the control group, and eight studies had no acupuncture treatment as the control. For treatment combination, one study compared three different interventions of acupuncture combined with bee venom, sham acupuncture, and no acupuncture [32]. Eleven of the studies were randomized controlled trials [8,19,20,22,23,28,30,31,32,34,35], and the other two were clinical trials [29,33]. The sample sizes ranged from 16 to 89 participants, with a mean age range of 55 to 75.4 years. Various scales were used to assess non-motor symptoms, including ESS (Epworth Sleepiness Scale); PDSS (Parkinson’s Disease Sleep Scale), PDSS-2 (Parkinson’s Disease Sleep Scale-2), PSQI (Pittsburgh Sleep Quality Index) for sleep disorder level, BDI (Beck’s Depression Inventory), BDI-II (Beck’s Depression Inventory-II), GDS (Geriatric Depression Scale), HADS-D (Hospital Anxiety and Depression Scale-Depression), HAM-D (Hamilton Depression Rating Scale), SDS (Self-Rating Depression Scale) for depression level, BAI (Beck Anxiety Inventory), HAM-A (Hamilton Anxiety Rating Scale), HADS-A (Hospital Anxiety and Depression Scale-Anxiety) for anxiety level, MFI-GF (Multidimensional Fatigue Inventory-General Fatigue), and MFIS (Modified Fatigue Impact Scale) for fatigue level. The intervention period of each study ranged from 30 days to 18 weeks.

### 3.3. Quality Assessment of Included Studies and Adverse Events

The 13 studies included in this meta-analysis underwent quality assessment using the modified Jadad scores, and the results are presented in Table 2. Among the 13 articles, 5 were rated as high quality [20,22,23,32,34], 5 had moderate quality [8,19,30,31,35], and 3 had low quality [28,29,33]. The studies with low-quality ratings had unclear reporting on randomization and blinding procedures, whereas studies with high-quality ratings implemented randomized methods and used sham acupuncture as a control to achieve double blinding. The overall quality of the literature was moderate, with an average score of 5.31. We also utilized the revised Cochrane risk-of-bias tool (RoB 2.0) to evaluate the risk of bias and quality of the included studies in the Supplementary Material (Figure S1). Furthermore, we conducted a comprehensive “Grading of Recommendations Assessment, Development, and Evaluation (GRADE)” assessment to evaluate the quality of evidence for our primary outcomes, as presented in the Supplementary Materials (Table S2). The assessment revealed that the level of certainty for sleep disorder outcomes was classified as low due to concerns regarding bias and inconsistent estimates. On the other hand, the level of certainty for depression outcomes was moderate, indicating consistent evidence in favor of acupuncture treatment and precise outcomes. Certainties for anxiety and fatigue outcomes were either very low or low, primarily due to limitations such as small sample sizes, imprecise outcomes, and potential bias. In addition, we have provided a comprehensive list of adverse events associated with acupuncture and electroacupuncture treatment in the Supplementary Materials (Table S3). Our findings indicate that both acupuncture and electroacupuncture are considered safe for the treatment of PD.

### 3.4. The Primary Outcome of Acupuncture in Sleep Disorders Improvement

**Figure 2:**
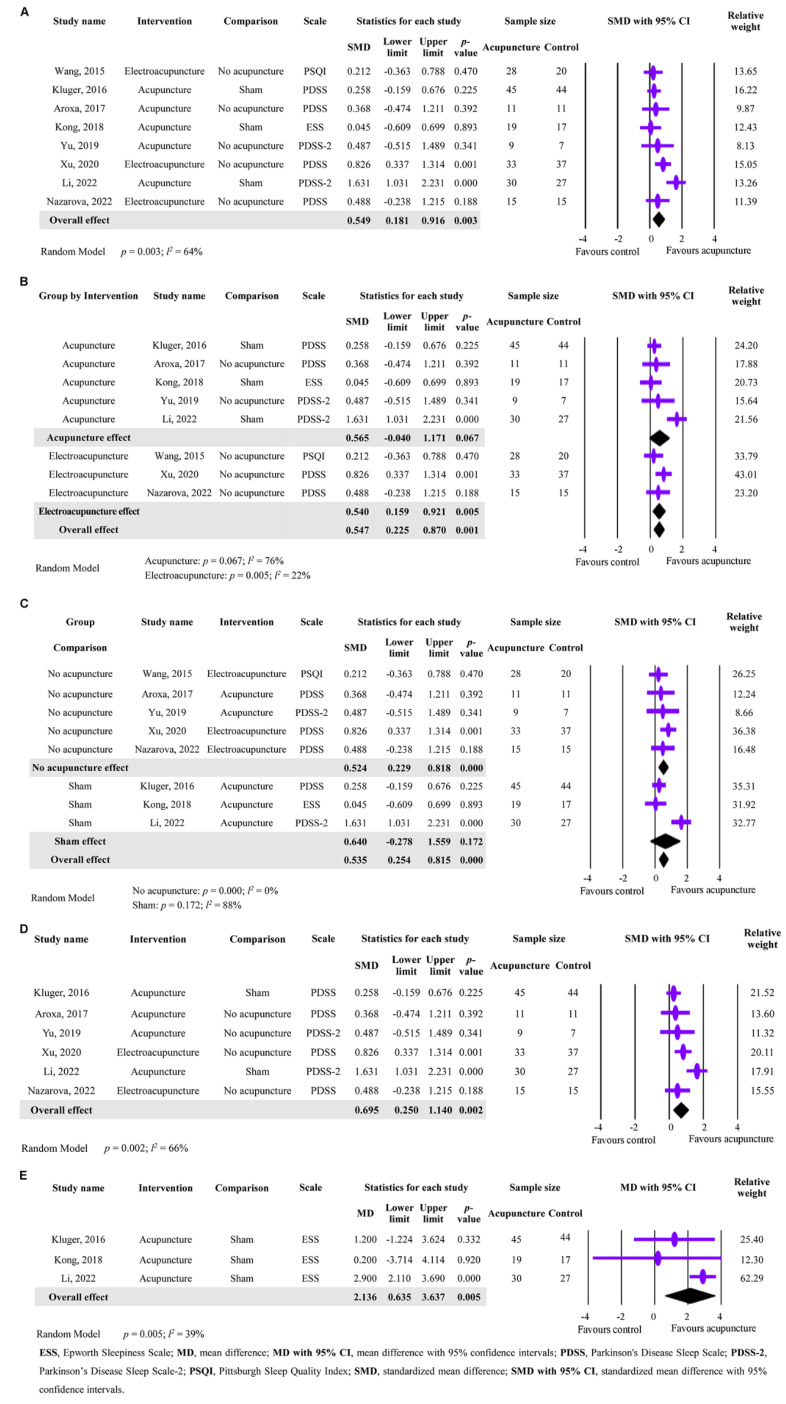
Meta-analysis of acupuncture’s effectiveness in improving sleep disorders in PD patients. (**A**) Forest plot showing the primary outcome of acupuncture’s effectiveness on sleep disorders. (**B**) Subgroup analysis of the effect of acupuncture intervention on sleep disorders based on the type of acupuncture (acupuncture or electroacupuncture). (**C**) Subgroup analysis of the effect of acupuncture intervention on sleep disorders based on different control groups (sham acupuncture or no acupuncture) (**D**) Subgroup analysis of the effect of acupuncture on sleep disorders using the scales of PDSS+PDSS-2. (**E**) Subgroup analysis of the effect of acupuncture on sleep disorders using the ESS scale [19,20,23,30,31,33,34,35].

The results of the analysis focused on the effectiveness of acupuncture in improving sleep disorders, which was the primary outcome, are presented in Figure 2. In total, eight studies involving 368 participants were included in the meta-analysis, and a significant difference was found between the acupuncture and control groups, despite substantial heterogeneity being observed (SMD = 0.549; 95% CI: 0.181 to 0.916; *p* = 0.003; *I*^2^ = 64%; Figure 2A). This indicates that acupuncture intervention can improve sleep disorders in PD patients. For the heterogeneity, a subgroup analysis was conducted based on the type of acupuncture intervention. As shown in Figure 2B, acupuncture showed a limited effect on improving sleep disorders (SMD = 0.565; 95% CI: −0.040 to 1.171; *p* = 0.067; *I*^2^ = 76%), while electroacupuncture showed a significant improvement (SMD = 0.540, 95% CI: 0.159 to 0.921, *p* = 0.005; *I*^2^ = 22%). The results show that electroacupuncture was more effective than acupuncture in improving sleep disorders in PD patients (Figure 2B). For further analysis, we compared the acupuncture techniques, including the acupoints and treatment protocols, as shown in the Supplementary Material (Table S4). Interestingly, our findings reveal an intriguing pattern: the GB20 (Fengchi) acupoint was consistently utilized in three out of the four electroacupuncture studies examined (Table S4). Figure 2C presents the subgroup analysis of the effect of acupuncture on sleep disorders in PD patients according to different control groups, namely sham acupuncture or no acupuncture. The results show that both acupuncture and electroacupuncture significantly improved sleep disorders in PD patients when compared to no acupuncture (SMD = 0.524; 95% CI: 0.229 to 0.818, *p* = 0.000; *I*^2^ = 0%). However, when compared to sham acupuncture, acupuncture only produced a limited effect on sleep disorders (SMD = 0.640, 95% CI: −0.278 to 1.559; *p* = 0.172; *I*^2^ = 88%). These findings suggest that acupuncture or electroacupuncture is effective in improving sleep disorders in PD patients when compared to no acupuncture, and that sham acupuncture may have a placebo effect on sleep disorders. Another analysis was conducted to assess the effect of acupuncture on sleep quality using different scales, namely PDSS+PDSS-2 or ESS. The results of six studies that used PDSS or PDSS-2 show a significant difference with substantial heterogeneity between the acupuncture and control groups (SMD = 0.695, 95% CI: 0.250 to 1.140; *p* = 0.002; *I*^2^ = 66%) (Figure 2D). The results of three studies that used ESS also show a significant difference with no substantial heterogeneity between the acupuncture and control groups (MD = 2.136, 95% CI: 0.635 to 3.637; *p* = 0.005; *I*^2^ = 39%) (Figure 2E). Overall, these findings demonstrate that acupuncture or electroacupuncture can effectively improve sleep disorders in PD patients when evaluated by the PDSS, PDSS-2, or ESS scales, and the type of acupuncture intervention and control group may affect the magnitude of the effect.

### 3.5. The Secondary Outcome of Acupuncture in Depression Improvement

**Figure 3:**
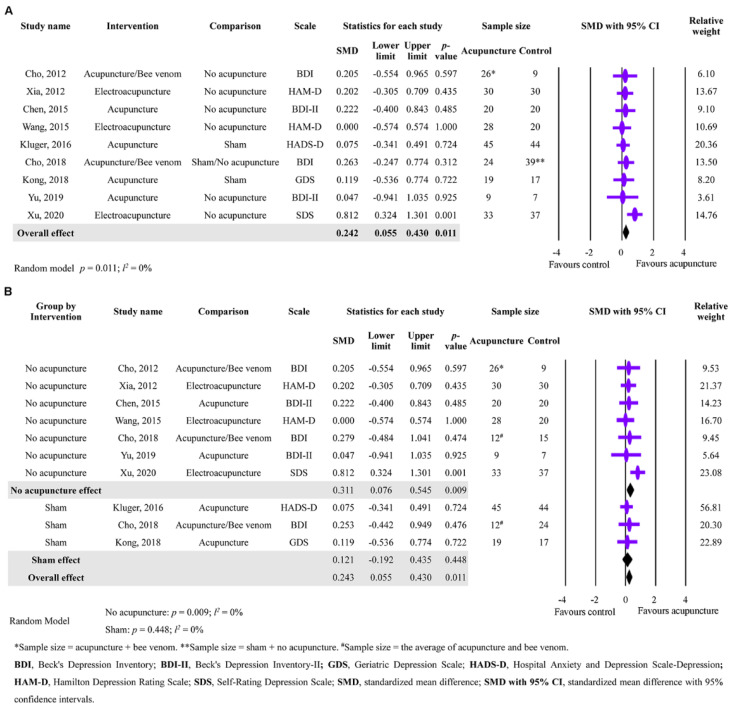
Meta-analysis of acupuncture’s effectiveness in improving depression in PD patients. (**A**) Forest plot showing the second outcome of acupuncture’s effectiveness on depression. (**B**) Subgroup analysis of the effect of acupuncture intervention on depression based on different control groups (sham acupuncture or no acupuncture) [8,20,23,28,29,30,32,33,34].

Acupuncture’s effect on depression improvement was investigated in this study’s secondary outcomes. Nine studies with a total of 457 participants were analyzed, and the results indicated a significant difference with no substantial heterogeneity between acupuncture and the control group, suggesting that acupuncture significantly improved depression (SMD = 0.242; 95% CI: 0.055 to 0.430; *p* = 0.011; *I*^2^ = 0%) (Figure 3A). A further analysis was conducted on the effect of different control groups (no acupuncture or sham), and it was found that acupuncture significantly improved depression in PD patients compared to no acupuncture (SMD = 0.311; 95% CI: 0.076 to 0.545; *p* = 0.009; *I*^2^ = 0%). Nevertheless, compared to sham acupuncture, acupuncture did not improve depression in PD patients (SMD = 0.121; 95% CI: −0.192 to 0.435; *p* = 0.448; *I*^2^ = 0%). These findings suggest that acupuncture, acupuncture/bee venom, or electroacupuncture may have a small effect on improving depression in PD patients compared to no acupuncture, and sham acupuncture may have a placebo effect on depression. 

### 3.6. The Secondary Outcomes of Acupuncture in Anxiety and Fatigue Improvement

**Figure 4:**
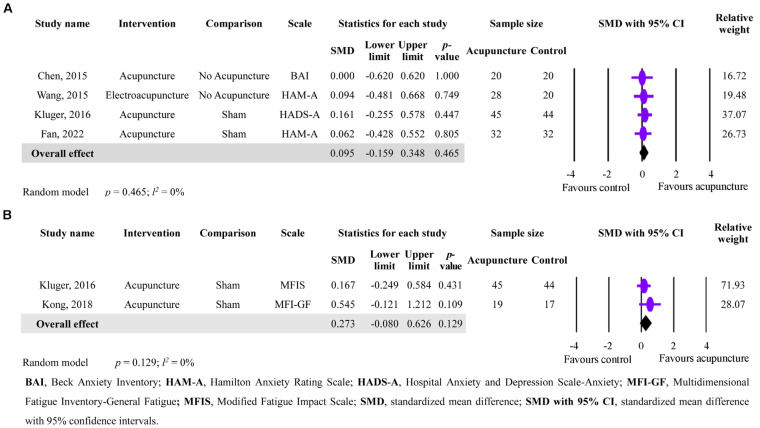
Forest plot showing the secondary outcomes of acupuncture in anxiety and fatigue improvement in PD patients. (**A**) Effects of acupuncture on anxiety improvement. (**B**) Effects of acupuncture on fatigue improvement [20,22,23,29,30].

In terms of acupuncture’s effect on anxiety and fatigue improvement, the analysis of four studies with a total of 241 participants with anxiety and two studies with a total of 125 participants with fatigue revealed no significant effect compared to the control group (anxiety: SMD = 0.095; 95% CI = −0.159 to 0.348; *p* = 0.465; *I*^2^ = 0%; fatigue: SMD = 0.273; 95% CI = −0.080 to 0.626; *p* = 0.129; *I*^2^ = 0%). Therefore, acupuncture did not show any improvements in anxiety and fatigue in PD patients (Figure 4A,B).

## 4. Discussion

This meta-analysis aimed to assess the effectiveness of acupuncture as a treatment for non-motor symptoms of Parkinson’s disease (PD). The study began by conducting a comprehensive search of various databases, yielding 1821 articles for screening. After eliminating articles that did not meet the inclusion criteria, 13 studies were selected for analysis, consisting of 630 PD patients aged between 55 and 75.4 years. Non-motor symptoms of sleep disorders, depression, anxiety, and fatigue were evaluated using various scales, with sleep disorders as the primary outcome and depression, anxiety, and fatigue as the secondary outcomes. The modified Jadad scores rated the overall quality of the literature as moderate. Results from this meta-analysis revealed that acupuncture intervention significantly improved sleep disorders in PD patients compared to control groups. Additionally, electroacupuncture was found to be more effective than traditional acupuncture, while sham acupuncture showed a potential placebo effect. Acupuncture was found to have a small effect on depression, while showing no significant improvements in anxiety and fatigue in PD patients compared to the control group. The differential effectiveness of acupuncture on sleep and depression, as compared to anxiety and fatigue, may be attributed to the specific utilization of electroacupuncture for PD-related sleep and depression, while its application for anxiety and fatigue was not explored. It is important to note that the analysis included a limited number of studies, with only four and two studies available for analyzing anxiety and fatigue, respectively, whereas eight and nine studies were included for investigating sleep disorders and depression, respectively. Moreover, a study by Wang et al. (2015) offers evidence supporting the mechanism behind electroacupuncture’s impact on sleep quality and depression, which involves an increase in norepinephrine levels and anti-neuroinflammation by reducing the elevation of nitric oxide levels [30].

Sleep disorders are a common non-motor symptom of PD, with their frequency increasing as the disease progresses [15]. Sleep disorders can have a negative impact on patients’ quality of life, mood, daytime function, and sleep quality [15]. Depression is also prevalent in PD patients, with an incidence of around 30% [36]. For patients experiencing obstructive sleep apnea (OSA), palate surgery has shown potential benefits in reducing the apnea and hypopnea index, daytime sleepiness, and associated mood-related comorbidities [37]. Surgical techniques such as lateral pharyngoplasty (LP) and uvulopalatopharyngoplasty (UPPP) have demonstrated effectiveness in treating OSA in adults [38]. A recent meta-analysis found that combining acupuncture with medication may be more effective in improving PD-related insomnia and depression than medication alone [21]. The report found that eight weeks may be the optimal course for improving PD-related insomnia [21]. Based on our research findings, electroacupuncture has shown superior efficacy in improving sleep disorders among PD patients. Taking into account the detailed summary of acupoints and treatment protocols from each study (Table S4), we propose a specific treatment protocol involving electroacupuncture targeting the GB20 (Fengchi) acupoint. This protocol suggests using continuous waves at a frequency of 100 Hz for a duration of 30 mins. We recommend administering this treatment protocol 2 to 4 times per week for a period of 8 weeks. However, further clinical trials are necessary to validate the efficacy of our electroacupuncture protocol. 

As for anxiety, Fan et al. (2022) reported that real acupuncture for 8 weeks does not improve PD-related anxiety at the end of treatment as compared with sham acupuncture [22]. However, real acupuncture shows an improvement in anxiety during the 8-week follow-up period [22]. Our meta-analysis also showed that acupuncture did not have a significant effect on anxiety at the end of treatment as compared to no acupuncture and sham. Additionally, Kong et al. (2018) reported that both real and sham acupuncture improve PD-related fatigue [23]. However, our meta-analysis showed that acupuncture did not have a significant effect on fatigue compared to the control group. This may be due to our meta-analysis only including four and two studies for anxiety and fatigue, respectively. In conclusion, our results support the use of acupuncture to improve PD-related sleep disorders and depression, but further research is necessary to investigate the potential interplay between acupuncture, sham acupuncture, and PD medication. 

Our study has the following limitations: (1) Limited number of studies: The meta-analysis only included a limited number of studies, comprising eight studies for the primary outcome of sleep disorders, and nine, four, and two studies for the secondary outcomes of depression, anxiety, and fatigue, respectively. This relatively small pool of studies may constrain the generalizability of our findings; for instance, important factors such as age and gender, which can influence the clinical course of PD. However, the results of our meta-regression analysis revealed that age and gender did not demonstrate statistically significant impacts on the outcomes of acupuncture in PD-related sleep disorders and depression. (2) Lack of blinding: Most of the studies included in the meta-analysis did not use blinding, which may introduce bias and affect the validity of the findings. (3) Lack of long-term follow-up: Most of the studies included in the meta-analysis had a short follow-up period, ranging from 4 to 12 weeks. Therefore, it is unclear whether the improvements in sleep disorders, depression, anxiety, and fatigue are sustained over a longer period. Factors such as the age at disease onset, disease severity and duration, PD subtype, and medical treatments can influence PD-related sleep disorders, depression, anxiety, and fatigue [39,40]. However, the lack of direct comparisons between these PD-related factors hinders our ability to assess the specific effects of acupuncture on these outcomes in the present study. Therefore, conducting additional well-designed clinical trials that carefully consider these PD-related factors is essential to validate and strengthen our findings.

In summary, our meta-analysis has demonstrated that acupuncture can be an effective treatment option for non-motor symptoms of sleep disorders and depression in PD patients. However, the evidence for PD-related anxiety and fatigue is currently limited, and further high-quality studies are needed to confirm the findings. PD is a neurodegenerative disorder with no known cure; various therapies and medications can improve its symptoms. Acupuncture is therapeutically effective for motor symptoms and provides neuroprotection by preventing the death of dopaminergic neurons. It is important to note that non-motor symptoms tend to worsen in severity and diversity as PD progresses. Therefore, PD patients experiencing anxiety and fatigue may consider acupuncture as a potential intervention to prevent further deterioration of their motor condition. Acupuncture is a commonly used alternative therapy for PD patients, and our findings suggest that it offers dual benefits for both motor and non-motor symptoms. Consequently, healthcare professionals may consider acupuncture as a viable treatment option for PD patients. Furthermore, our study results can provide valuable guidance for the clinical application of acupuncture in treating PD.

## Figures and Tables

**Table 1:** Characteristics of studies included in the meta-analysis.

Study	Country	Study Design	Comparison	Subjects (N)	Age Mean ± SD	Diagnostic Criteria for Non-Motor Symptoms	Treatment Duration	Population
Cho et al., 2012 [8]	Korea	Randomized controlled trial	Acupuncture Bee venom No acupuncture	13 13 9	55.0 (52.0, 66.0) * 57.0 (49.0, 69.0) * 57.0 (48.0, 68.0) *	BDI, PDQL	8 weeks	Idiopathic PD
Xia et al., 2012 [28]	China	Randomized controlled trial	Electroacupuncture No acupuncture	30 30	72 ± 7 72 ± 8	HAM-D, BDNF	3 months	PD with depression
Chen et al., 2015 [29]	Taiwan	Clinical trial	Acupuncture No acupuncture	20 20	72.1 ± 8.5 75.4 ± 12.2	BAI, BDI-II, WHOQOL	18 weeks	PD
Wang et al., 2015 [30]	China	Randomized controlled trial	Electroacupuncture No acupuncture	28 20	62.1 ± 8.7 59.1 ± 12.4	NMSQ, MoCA, MMSE, HAM-D, HAM-A, PSQI, PDQ-39	2 months	PD
Kluger et al., 2016 [20]	USA	Randomized controlled trial	Acupuncture Sham	45 44	64.4 ± 10.3 63.0 ± 13.0	MFIS, PDSS, ESS, HADS-A, HADS-D, PDQ-39	6 weeks	PD
Aroxa et al., 2017 [31]	Brazil	Randomized controlled trial	Acupuncture No acupuncture	11 11	65 ± 10 56 ± 12	PDSS, MMSE	8 weeks	Idiopathic PD
Cho et al., 2018 [32]	Korea	Randomized controlled trial	Acupuncture + Bee venom Sham Conventional treatment	24 24 15	64.42 ± 8.24 61.33 ± 8.20 64.07 ± 6.33	BDI, PDQL	12 weeks	Idiopathic PD
Kong et al., 2018 [23]	Singapore	Randomized controlled trial	Acupuncture Sham	19 17	66.4 ± 6.5 62.9 ± 9.7	ESS, GDS, MFI-GF, PDQ-39	5 weeks	PD
Yu et al., 2019 [33]	Taiwan	Clinical trial	Acupuncture No acupuncture	9 7	60.7 ± 6.3 70.4 ± 8.2	PDSS-2, BDI-II, MMSE, KPPS, VAS, PDQ-39	8 weeks	Idiopathic PD
Xu et al., 2020 [34]	China	Randomized controlled trial	Electroacupuncture No acupuncture	33 37	61.73 ± 10.28 61.95 ± 9.77	PDSS, SDS	8 weeks	PD
Fan et al., 2022 [22]	China	Randomized controlled trial	Acupuncture Sham	32 32	61.03 ± 9.80 62.66 ± 6.94	HAM-A, PDQ-39	8 weeks	Idiopathic PD
Li et al., 2022 [19]	China	Randomized controlled trial	Acupuncture Sham	30 27	63 ± 6.73 59 ± 9.28	PDSS-2, ESS	30 days	PD
Nazarova et al., 2022 [35]	China	Randomized controlled trial	Electroacupuncture No acupuncture	15 15	70.1 ± 6.2 66.9 ± 7.8	PDSS, NMSS, BSFS, PAC-QOL	8 weeks	PD

* Data are expressed by median (lower quartile, upper quartile). BAI, Beck Anxiety Inventory; BDI, Beck’s Depression Inventory; BDI-II, Beck’s Depression Inventory-II; BDNF, Brain-Derived Neurotrophic Factor; BSFS, Bristol Stool Form Scale; ESS, Epworth Sleepiness Scale; GDS, Geriatric Depression Scale; HADS-A, Hospital Anxiety and Depression Scale-Anxiety; HADS-D, Hospital Anxiety and Depression Scale-Depression; HAM-A, Hamilton Anxiety Rating Scale; HAM-D, Hamilton Depression Rating Scale; KPPS, King’s Parkinson’s Disease Pain Scale; MFI-GF, Multidimensional Fatigue Inventory-General Fatigue; MFIS, Modified Fatigue Impact Scale; MMSE, Mini-Mental State Examination; MoCA, Montreal Cognitive Assessment; NMSQ, Non-Motor Symptoms Questionnaire; NMSS, Non-Motor Symptoms Scale; PAC-QOL, Patient Assessment of Constipation Quality of Life questionnaire; PD, Parkinson’s disease; PDQ-39, Parkinson’s Disease Questionnaire; PDQL, Parkinson’s Disease Quality of Life Questionnaire; PDSS, Parkinson’s Disease Sleep Scale; PDSS-2, Parkinson’s Disease Sleep Scale-2; PSQI, Pittsburgh Sleep Quality Index; SDS, Self-Rating Depression Scale; WHOQOL, World Health Organization Quality of Life; VAS, Visual Analogue Scales.

**Table 2:** Quality of the included studies by Modified Jadad scores.

Study	Was the Study Described as Randomized? (Yes: 1; No: 0)	Was the Method of Randomization Appropriate? (Yes: 1; No: −1; Not Described: 0)	Was the Study Described as Blinding? (Yes: 1; No: 0)	Was the Method of Blinding Appropriate? (Yes: 1; No: −1; Not Described: 0)	Was There a Description of Withdrawals and Dropouts? (Yes: 1; No: 0)	Was There a Clear Description of the Inclusion and Exclusion Criteria? (Yes: 1; No: 0)	Was the Method Used to Assess Adverse Effects Described? (Yes: 1; No: 0)	Was the Methods of Statistical Analysis Described? (Yes: 1; No: 0)	Total Scores
Cho et al., 2012 [8]	1	0	0	0	1	1	1	1	5
Xia et al., 2012 [28]	1	−1	0	0	1	1	0	1	3
Chen et al., 2015 [29]	0	0	0	0	0	1	1	1	3
Wang et al., 2015 [30]	1	0	0	0	1	1	0	1	4
Kluger et al., 2016 [20]	1	1	1	1	1	1	1	1	8
Aroxa et al., 2017 [31]	1	0	0	0	1	1	0	1	4
Cho et al., 2018 [32]	1	1	1	1	1	1	1	1	8
Kong et al., 2018 [23]	1	1	1	1	1	1	1	1	8
Yu et al., 2019 [33]	0	0	0	0	0	1	0	1	2
Xu et al., 2020 [34]	1	1	1	0	1	1	1	1	7
Fan et al., 2022 [22]	1	1	1	1	1	1	1	1	8
Li et al., 2022 [19]	1	1	0	0	0	1	0	1	4
Nazarova et al., 2022 [35]	1	1	0	0	0	1	1	1	5

The total score for each article ranged from 0 to 8; a score of 7 or 8 was considered to be of high quality, 4–6 of moderate quality, and 1–3 of low quality.

## Data Availability

The datasets used and/or analyzed during the current study are available from the corresponding authors upon reasonable request.

## Supplementary Materials

The following supporting information can be downloaded at: https://www.mdpi.com/article/10.3390/healthcare11142042/s1, Figure S1: RoB-2 evaluation of included studies; Table S1: Literature search strings for our meta-analysis in various databases; Table S2: Grading of Recommendations Assessment, Development, and Evaluation (GRADE) evidence profile for the included studies; Table S3: Adverse events associated with acupuncture and electroacupuncture in each study; Table S4: A detailed summary of the acupoints and treatment protocol in each study.
