# Supplementary material for: Acupuncture Improves Sleep Disorders and Depression among Patients with Parkinson’s Disease: A Meta-Analysis
Source: Healthcare (Basel). 2023 Jul 17;11(14):2042. doi: 10.3390/healthcare11142042 (PMC10379076; doi:10.3390/healthcare11142042)
Supplement: Supplementary file 1 [file healthcare-11-02042-s001.zip › healthcare-2457975-supplementary.pdf]

Supplementary Material: Acupuncture Improves Sleep Disorders and Depression among Patients with Parkinson's Disease: A Meta-Analysis

Wei-Ti Hsu, Chieh-Min Hsu, Shao-Chi Hung and Shih-Ya Hung

Table S1. Literature search strings for our meta-analysis in various databases.

Database	Strings
Pubmed	((Parkinson disease) OR Parkinsonism) AND (acupuncture OR electroacupuncture)
Embase	('parkinson disease'/exp OR 'parkinson disease' OR (parkinson AND ('disease'/exp OR disease)) OR 'parkinsonism'/exp OR parkinsonism) AND ('acupuncture'/exp OR acupuncture OR 'electroacupuncture'/exp OR electroacupuncture)
Web of Science	((Parkinson disease) OR Parkinsonism) AND (acupuncture OR electroacupuncture)
CINAHL	((Parkinson disease) OR Parkinsonism) AND (acupuncture OR electroacupuncture)

Table S2. Grading of Recommendations Assessment, Development, and Evaluation (GRADE) evidence profile for the included studies.

Certainty assessment											
Outcome	No. of trials	Study design	Risk of bias	Inconsistency	Indirectness	Imprecision	Other	Number of patients (n)		Effect estimate (95% CI)	Quality of evidence
								Acupuncture	Control		
Sleep disorders	8	RCTs	Serious*	Serious**	Not serious	Not serious	None	190	178	SMD 0.549 (0.181 to 0.916)	⊕⊕◯◯ Low
Depression	9	RCTs	Serious*	Not serious	Not serious	Not serious	None	234	223	SMD 0.242 (0.055 to 0.430)	⊕⊕⊕◯ Moderate
Anxiety	4	RCTs	Serious*	Not serious	Not serious	Serious***	None	125	116	SMD 0.095 (-0.159 to 0.348)	⊕◯◯◯ Very low
Fatigue	2	RCTs	Not serious	Not serious	Not serious	Serious***	None	64	61	SMD 0.273 (-0.080 to 0.626)	⊕⊕◯◯ Low

GRADE: Grading of Recommendations, Assessment, Development, and Evaluations
RCT: Randomized controlled trials; CI: Confidence interval; SMD: Standard Mean difference.
*The modified Jadad score of included articles indicated moderate quality.
**Substantial heterogeneity (I square > 50%).
***95% Confidence interval includes both acupuncture and Control (p > 0.05).

Table S3. Adverse events associated with acupuncture and electroacupuncture in each study.

Study	Adverse events
Cho et al., 2012 [8]	One subject in the bee venom acupuncture group complained of itchiness.
Xia et al., 2012 [28]	Not available
Chen et al., 2015 [29]	There were no side effects or serious adverse events.
Wang et al., 2015 [30]	Not available
Kluger et al., 2016 [20]	Increased constipation in one participant receiving real acupuncture
Aroxa et al., 2017 [31]	Not available
Cho et al., 2018 [32]	No serious adverse events were noted during the study period. Only mild itchiness or mild swelling after bee venom acupuncture.
Kong et al., 2018 [23]	A total of three adverse events were reported. But, all adverse events were deemed not related to acupuncture treatment.
Yu et al., 2019 [33]	Not available
Xu et al., 2020 [34]	No acupuncture-related adverse events were observed in either group.
Fan et al., 20229 [22]	Four mild adverse reactions occurred during the study. However, no serious adverse events occurred.
Li et al., 2022 [19]	Not available
Nazarova et al., 2022 [35]	One patient from the electroacupuncture group reported two recent falls during the treatment period.

Table S4. A detailed summary of the acupoints and treatment protocol in each study.

Study	Acupuncture or electroacupuncture	Acupoints	Treatment Protocol
Cho et al., 2012 [8]	Acupuncture	GB20 (Fengchi), LI11 (Quchi), GB34 (Yanglingquan), ST36 (Zusanli), LR3 (Taichong)<br>*Bilateral	20 mins; twice per week for 8 weeks
Xia et al., 2012 [28]	Electroacupuncture	GV20 (Baihui), EX-HN3 (Yintang), EX-HN1 (Sishencong), LR3 (Taichong), SP6 (Sanyinjiao)	30 mins; once every 2 days for 3 months
Chen et al., 2015 [29]	Acupuncture	GV20 (DU20, Baihui), GB20 (Fengchi), LI11 (Quchi), LI10 (Shousanli), LI4 (Hegu), GB31 (Fengshi), ST32 (Futu), GB34 (Yanglingquan), GB38 (Yangfu)<br>*Bilateral except DU20	>15 mins; twice per week for 18 weeks
Wang et al., 2015 [30]	Electroacupuncture	GB20 (Fengchi), LI4 (Hegu), Du14 (Dazhui), Du16 (Fengfu)<br>*Bilateral	Electrical pulses of 9 V, 1 A, 9 W, and 100 Hz for 30 mins; once every 3 days for 2 months
Kluger et al., 2016 [20]	Acupuncture	GV20 (Baihui), GV24 (Shenting), LI10 (Shousanli), HT7 (Shenmen), ST36 (Zusanli), SP6 (Sanyinjiao)	30 mins; twice per week for 6 weeks
Aroxa et al., 2017 [31]	Acupuncture	LR3 (Taichong), SP6 (Sanyinjiao), LI4 (Hegu), TE5 (Waiguan), HT7 (Shenmen), PC6 (Neiguan), LI11 (Quchi), GB20 (Fengchi).	30 mins; once per week for 8 weeks
Cho et al., 2018 [32]	Acupuncture	GB20 (Fengchi), LI11 (Quchi), GB34 (Yanglingquan), ST36 (Zusanli), LR3 (Taichong)<br>*Bilateral	15 mins; twice per week for 12 weeks
Kong et al., 2018 [23]	Acupuncture	PC6 (Neiguan), LI4 (Hegu), ST36 (Zusanli), SP6 (Sanyinjiao), KI3 (Taixi), CV6 (Qihai).	20 mins; twice per week for 5 weeks
Yu et al., 2019 [33]	Acupuncture	GV20 (Baihui), Shen Guan (77.18), GB34 (Yanglingquan)	30 mins; one to three times per week for 8 weeks
Xu et al., 2020 [34]	Electroacupuncture	GV17 (Naohu), GB19 (Naokong), EX-HN 1 (Sishencong) and temporal three-needle, GV20 (Baihui)	Continuous waves at alternating low 100 Hz frequency; 30 mins; 4 times per week for 8 weeks
Fan et al., 20229 [22]	Acupuncture	GV24 (Shenting), GV29 (Yintang), HT7 (Shenmen), SP6 (Sanyinjiao), EX-HN 1 (Sishencong)	30 mins; 3 times per week for 8 weeks
Li et al., 2022 [19]	Acupuncture	GV24 (Shenting), GV20 (Baihui), KI6 (Zhaohai), Guanyuan (CV4)	30 mins; once per day for 30 days
Nazarova et al., 2022 [35]	Electroacupuncture	GV20 (Baihui), GB20 (Fengchi), CV4 (Guanyuan), CV12 (Zhongwan), ST25 (Tianshu), ST36 (Zusanli), SP-6 (Sanyinjiao), LI4 (Hegu), ST40 (Fenglong), LR3 (Taichong)	Continuous wave at 50/100 Hz; 30 mins; twice per week for 8 weeks

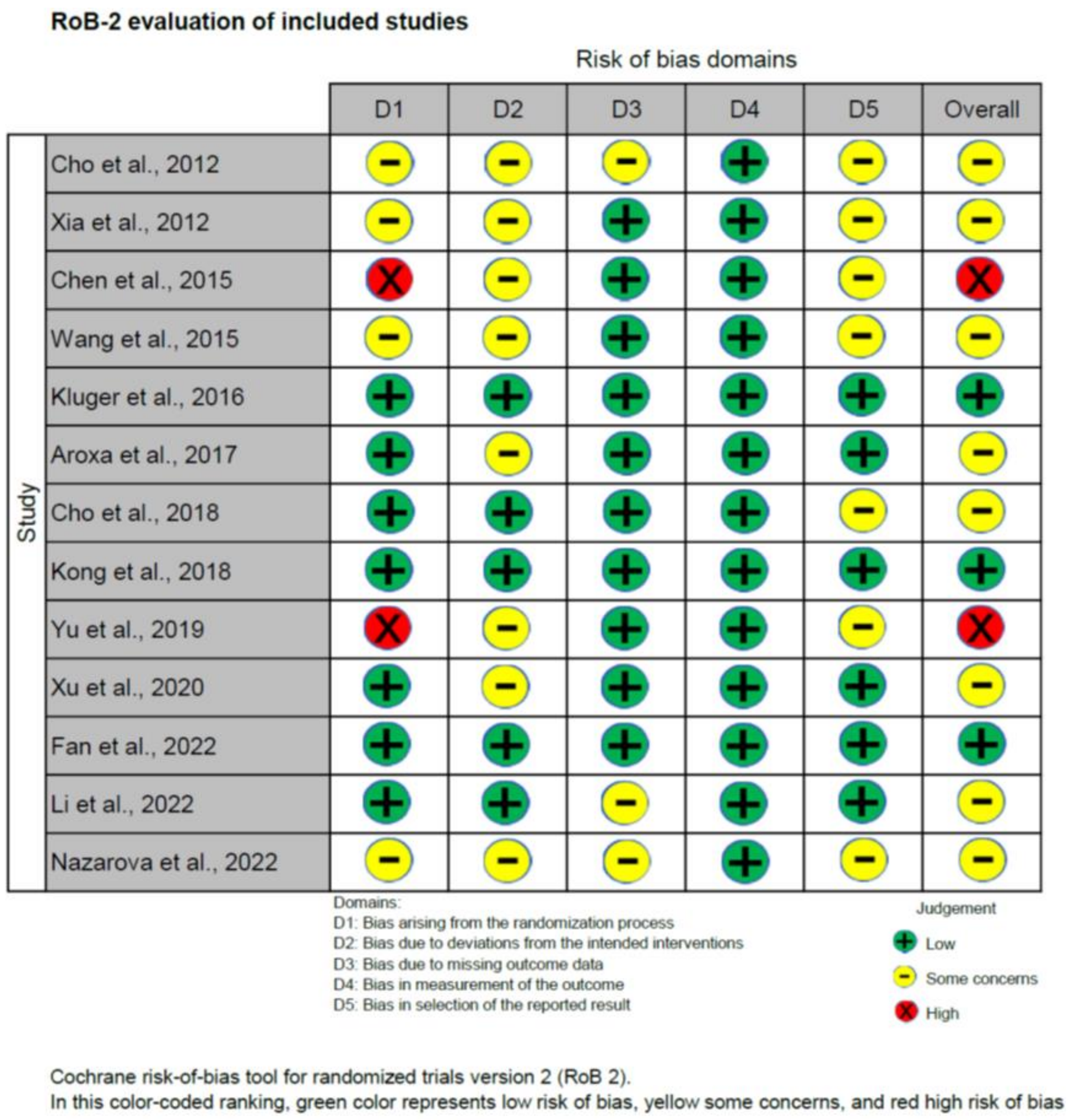

Figure S1. RoB-2 evaluation of included studies.
